# Compressive Mechanical Behavior and Corresponding Failure Mechanism of Polymethacrylimide Foam Induced by Thermo-Mechanical Coupling

**DOI:** 10.3390/polym16091199

**Published:** 2024-04-25

**Authors:** Zeyang Xing, Qianying Cen, Qingyou Wang, Lili Li, Zhigang Wang, Ling Liu

**Affiliations:** 1 School of Aerospace Engineering & Applied Mechanics, Tongji University, Shanghai 200092, China; 2130891@tongji.edu.cn (Z.X.); qianyingcen@tongji.edu.cn (Q.C.); 2 Avic Harbin Aircraft Industry Group Co., Ltd., Harbin 150066, China; wangqingyou2022@163.com (Q.W.); lilili@avic.com (L.L.); zhigangwang@163.com (Z.W.)

**Keywords:** PMI, foam, compression, failure mechanism

## Abstract

Thermal–mechanical coupling during the molding process can cause compressive yield in the polymer foam core and then affect the molding quality of the sandwich structure. This work investigates the compressive mechanical properties and failure mechanism of polymethacrylimide (PMI) foam in the molding temperature range of 20–120 °C. First, the DMA result indicates that PMI foam has minimal mechanical loss in the 20~120 °C range and can be regarded as an elastoplastic material, and the TGA curve further proves that the PMI foam is thermally stable within 120 °C. Then, the compression results show that compared with 20 °C, the yield stress and elastic modulus of PMI foam decrease by 22.0% and 17.5% at 80 °C and 35.2% and 31.4% at 120 °C, respectively. Meanwhile, the failure mode changes from brittle fracture to plastic yield at about 80 °C. Moreover, a real representative volume element (rRVE) of PMI foam is established by using Micro-CT and Avizo 3D reconstruction methods, and the simulation results indicate that PMI foam mainly shows brittle fractures at 20 °C, while both brittle fractures and plastic yield occur at 80 °C, and most foam cells undergo plastic yield at 120 °C. Finally, the simulation based on a single-cell RVE reveals that the air pressure inside the foam has an obvious influence of about 6.7% on the yield stress of PMI foam at 80 °C (brittle–plastic transition zone).

## 1. Introduction

Due to their excellent specific stiffness and thermal insulation properties, polymer foams have been widely used in aerospace, automotive, construction, etc. [1,2]. Polymer foams are often used as the core materials in composite sandwich structures [3,4,5,6]. Among them, polymethacrylimide (PMI) foam is a closed-cell and cross-linked rigid foam with the characteristics of high-temperature resistance, creep resistance, low thermal conductivity, and high heat-distortion-temperature [7,8] and has been increasingly used as the core material in aerospace composite sandwich structures [9,10,11]. However, during the molding process of sandwich structures, the PMI foam core is prone to compressive yield or collapse under high temperature and pressure, which may cause cracks in the core layer or debonding at the face-panel/core interface and ultimately affects the product quality [12]. Therefore, it is of great *engineering* significance to clarify the changing trend in the compressive mechanical properties of PMI foam and the corresponding failure mechanism during the molding process.

Currently, PMI foam mainly includes Rohacell WF, Rohacell IG, Rohacell HT, and Rohacell HERO. Among them, each series is further divided into multiple models based on density. For example, Rohacell WF can be primarily categorized into 51 WF, 71 WF, 110 WF, and 200 WF, with nominal densities of 52, 75, 110, and 205 kg/m^3^, respectively. Where Rohacell 51 WF exhibits a heat distortion temperature up to 200 °C [13], and the dimensional change rate in the range of 20–180 °C is only 0.58% [14], indicating that PMI foam has excellent thermal stability. In addition, Chen et al. [15] stated the compressive ultimate stress of PMI foam at room temperature is close to 1.2 MPa, and the stress–strain curve shows a “three-stage” pattern of the elastic section, plateau section, and densification section, in which the collapse zone first appears in the central section of foam sample and gradually expands around during the compression. Grace et al. [16] investigated the compressive response of PMI foam in the range of −60~22 °C and 0.127–12.7 mm/s, indicating that PMI foam becomes brittle and stiff with a decrease in temperature, and the stress in the plateau section increases with an increase in the loading rate. Siivola et al. [17] reported that the compressive ultimate stress of PMI foam is close to 0.9 MPa at 30 °C, which decreases by 30% at 80 °C, as well as some cavity walls exhibit brittle fracture at 30 °C. Meanwhile, Grenestedt et al. [18] pointed out that for closed-cell foam, the air pressure in the cell is roughly equal to the atmospheric pressure. Rusch et al. [19] indicated that when the foam matrix material is brittle, the air in the foam cavity will not affect the compressive performance of the foam, and when the foam matrix material is plastic, the influence of the air cannot be ignored. Previous studies have shown that the compressive ultimate stress of PMI foam is between 0.9 and 1.2 MPa, which significantly decreases with increasing temperature and increases with increasing loading rate. In addition, the gas in the foam cavity may also affect the overall compressive performance of the foam. However, although the existing research has reported the compressive behavior of PMI foam changing with temperature or strain rate, few studies have systematically explored its microscopic failure mechanism and the effect of air in foam cells at relatively high temperatures.

In fact, in order to better explore the microscopic failure mechanisms of foam, a real representative volume element (rRVE) based on the microstructure of foam should be established. Timpano et al. [20] constructed a 3D RVE for the polyvinylidene fluoride foam and obtained the volume/surface area of the foam cells by using Micro-CT technology. Ni et al. [21] obtained the compressive deformation characteristics and local failure behavior of copper foam based on Micro-CT. Su et al. [22] used Micro-CT to characterize the size and surface area of closed-cell aluminum foam and thus established a random 3D RVE based on the probability density function. Zhou et al. [23] obtained the geometric characteristics of closed-cell PVC foam based on Micro-CT and used the advancing front algorithm and Laguerre embedding algorithm to form the microstructure of foam. Chai et al. [24] combined Micro-CT with digital image correlation technology to characterize the cell volume change in PMI foam during compression and used the reconstructed foam microstructure (rRVE) for numerical simulation, suggesting that cell wall buckling occurred during the cell collapse. It can be seen from the aforementioned literature that the real microstructure characteristics of foam can be obtained through Micro-CT. However, in addition to Chai et al. [23], the existing studies rarely establish an rRVE for foam. In addition, the established rRVE by Chai et al. only contains three layers of foam cells. Bastawros et al. [25] pointed out that when too few foam cells are included in the model, the simulated stress will be significantly smaller than the experimental value. Thus, it is necessary to establish a reasonable rRVE to study the microscopic failure mechanism of PMI foam at different temperatures.

Therefore, this work studies the compressive mechanical behavior and microscopic failure mechanism of PMI foam within a molding temperature range of 20–120 °C. First, the dynamic mechanical analysis (DMA) method was used to explore the mechanical behaviors of PMI foam and PMI matrix material in the range of 20–120 °C. Second, the compressive mechanical properties and failure modes of PMI foam within 20–120 °C were experimentally investigated. Then, Micro-CT was used to characterize the microstructure of PMI foam and to establish a reasonable rRVE. Finally, the established rRVE was introduced into Abaqus for thermal–mechanical coupling analysis in order to explore the microscopic failure mechanisms of PMI foam at different temperatures, as well as the influence of air inside the foam cells on the compressive mechanical behavior of PMI foam.

## 2. Materials and Methods

### 2.1. Experimental Methods

**Figure 1:**
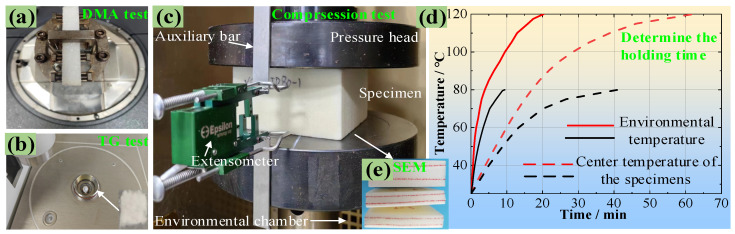
Experimental design, (**a**) DMA, (**b**) TG analysis, (**c**) Compression testing, (**d**) temperature-time curves of the environmental chamber and PMI specimen center, (**e**) SEM observation.

The used PMI foam was Rohacell 51WF, with an average density of 52 kg/m^3^. It is typically synthesized using the free radical prepolymerization method, utilizing methyl acrylonitrile and methyl acrylic acid as the primary monomers. The monomers are polymerized by an initiator at a low temperature to form a prepolymer, and then PMI foam is obtained by a foaming agent at a high temperature. First, to clarify whether the PMI foam/matrix is elastoplastic or viscoelastic, DMA tests (Figure 1a) were conducted on the PMI matrix (Single cantilever beam: 32 mm × 11 mm × 4 mm) and PMI foam (Three-point bending: 48 mm × 10 mm × 9 mm) according to ASTM D4065 [26] standard (Q800, TA Instruments, New Castle, DE, USA). The scanning temperature range was 25–200 °C, with a heating rate of 5 °C/min, a testing frequency of 1 Hz, and an amplitude of 25 μm. In addition, in order to prove that PMI foam is thermally stable, thermogravimetric analysis (TGA) experiments were conducted under a nitrogen atmosphere (209F1, NETZSCH, Selb, Germany), with a sample weight of 3~10 mg, a temperature range of 30~800 °C, and a heating rate of 10 °C/min, as given in Figure 1b. Both DMA and TGA experiments were repeated three times to ensure the reproducibility of these data.

Then, compression mechanical tests were performed on a universal testing machine with an environmental chamber (MTS-5T, MTS, Eden Prairie, MN, USA), and the specimen size is 100 mm × 100 mm × 50 mm according to ASTM D1621 standard [27], as shown in Figure 1c. Due to the low thermal conductivity of PMI foam, its center temperature is lower than the real-time temperature of the environmental chamber during the heating process. Therefore, a preliminary experiment was conducted. That is, a thermometer was inserted into the center of the PMI foam specimen, and a constant temperature operation at the measuring temperature point was performed; thereby, the time when the center temperature of the PMI foam reached the temperature of the environmental chamber can be obtained. Figure 1d illustrates the time required for the environmental chamber and the specimen center to reach 80 and 120 °C (The same method is used for other test temperatures), respectively. It shows that it will take 10 min and 20 min for the environmental chamber to reach 80 and 120 °C, respectively, while the PMI foam center requires 40 min and 63 min to reach 80 and 120 °C, respectively. Therefore, a constant temperature time of 30 min and 43 min is required for loading at 80 and 120 °C to ensure a uniform temperature distribution throughout the entire PMI specimen.

It was worth noting that during the heating/holding process, the reading of the force sensor was always kept at zero by automatically adjusting the loading head, thereby eliminating the influence of thermal stress. The testing temperatures were set as 20, 40, 60, 80, 100, and 120 °C, respectively. Upon reaching the loading temperature and after the holding time, a loading rate of 2 mm/min (strain rate is 0.00067/s) was applied to the specimen, where the deformation was collected by an extensometer (Epsilon, Irving, TX, USA), as shown in Figure 1a. It is worth noting that the number of specimens for each test temperature is 5–6 to ensure the reproducibility of the test results. After the testing, some damaged specimens (see Figure 1e) at different temperatures were characterized using an SEM machine (FESEM, MIRA3TESCAN, Brno, Czech Republic) to observe the failure modes.

### 2.2. Numerical Simulation Methods

#### 2.2.1. 3D Reconstruction of PMI Foam

**Figure 2:**
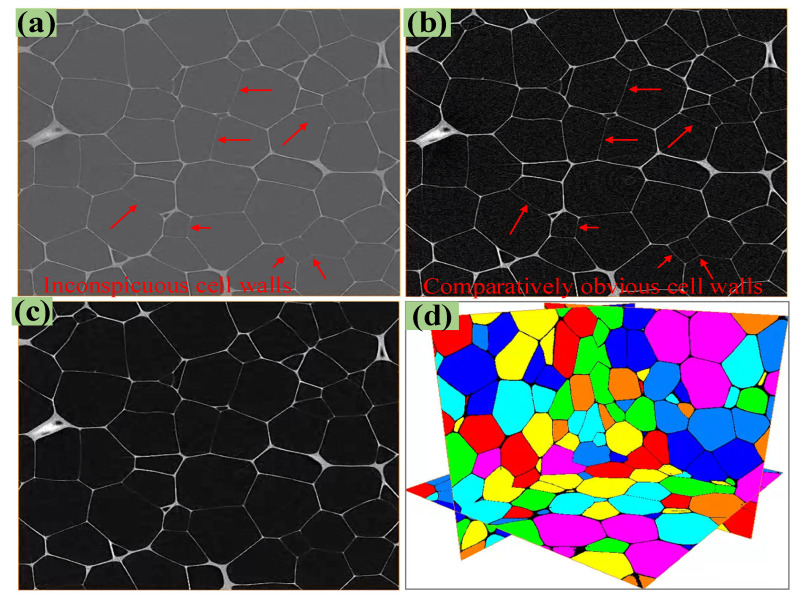
Processing of PMI foam Micro-CT slices, (**a**) initial slices with inconspicuous cell walls, (**b**) contrast adjustment with comparatively obvious cell walls, (**c**) nonlocal median filtering, (**d**) foam cell slice.

A 10 mm × 10 mm × 10 mm PMI foam specimen was used for Micro-CT tomography (YXLON-FF85CT, COMET, Hamburg, Germany), where the operating voltage, power, and spatial resolution used for the scanning were 90 kV, 250 W, and 3 μm, respectively. After scanning, a total of 3300 2D grayscale slices with a thickness of 1 pixel was obtained. In order to ensure a sufficient number of foam cells and also to improve the computational efficiency of subsequent rRVE, this work selected 1500 continuous 2D slices and intercepted 1500 pixels in both directions of each slice in-plane. Consequently, an rRVE model with actual dimensions of 4.482 mm × 4.482 mm × 4.482 mm was obtained. Due to the low Micro-CT value of PMI matrix material, the Micro-CT grayscale slices of local PMI foam are not obvious, as shown in Figure 2a. Thus, Avizo-2019 3D image processing software was used to adjust the contrast of the slices (in Figure 2a) to enhance the visibility of the thinner foam cavity walls, as given in Figure 2b, but which would increase the image noise. Therefore, the nonlocal mean filter in Avizo was applied to denoise the slices, and the resulting slices were given in Figure 2c. Finally, the threshold value of the foam cavity was set to 1, and the threshold value of the PMI matrix material was set to 0, thus establishing the PMI foam cell slice, as depicted in Figure 2d.

**Figure 3:**
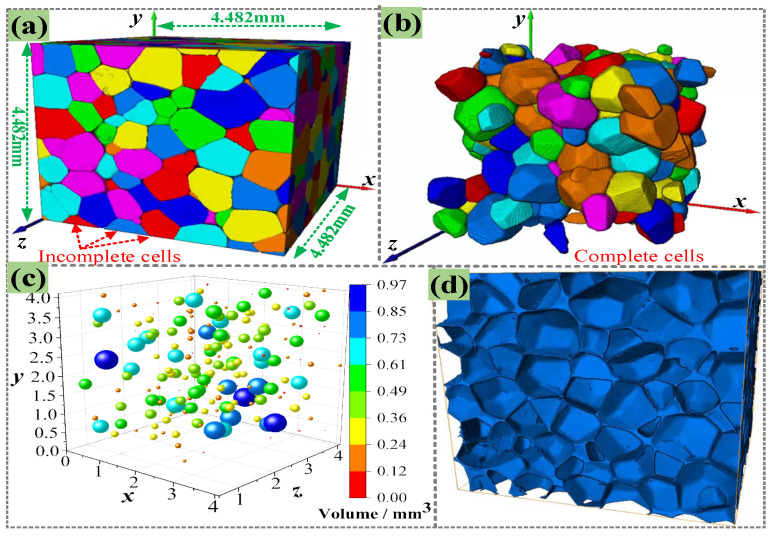
(**a**) 3D reconstruction model of PMI foam, (**b**) 3D reconstruction model only containing complete cells, (**c**) distribution of cell positions and sizes, (**d**) real microstructure of PMI foam (rRVE).

By assembling the 1500 continuous 2D slices, a 3D image of PMI foam was obtained, as given in Figure 3a, which shows that the foam cells on the boundaries are incomplete. Thus, all incomplete cells should be removed, as shown in Figure 3b. In addition, during the reconstruction of the 3D model, a threshold of 500 μm^3^ was set to exclude potential defects. Then, the centroid position and size of each complete foam cell were counted; thus, the size and location distribution of foam cells were obtained, as presented in Figure 3c. Finally, Boolean subtraction was performed on the 3D model, and the foam cell model was deleted from the entire 3D space to obtain the real microstructure of PMI foam, namely the rRVE, as shown in Figure 3d.

#### 2.2.2. Simulations Based on rRVE

**Figure 4:**
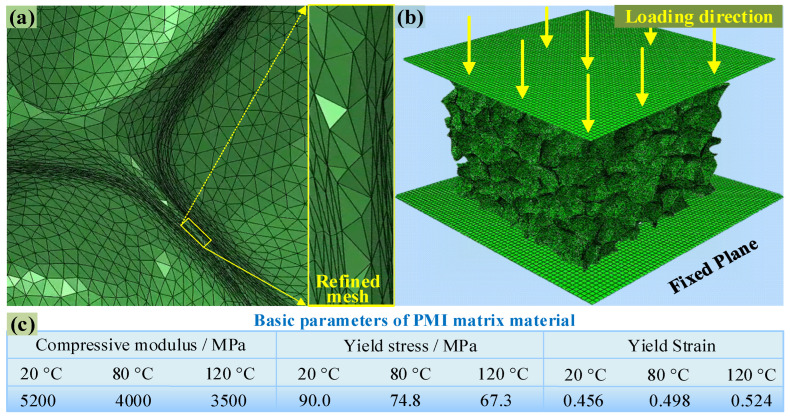
(**a**) Grid partitioning of rRVE, (**b**) finite element model for compression analysis, (**c**) basic parameters of PMI matrix material.

Jeon et al. [28] pointed out that when the number of foam cells contained in any direction exceeds five, the macro-mechanical properties of foam will no longer change with an increase in the number of foam cells. According to the 3D rRVE with a size of 4.482 mm × 4.482 mm × 4.482 mm shown in Figure 3d, each direction contains more than five complete cells, while the entire rRVE model contains a total of 206 PMI cells. First, extract the inner and outer surfaces of the 3D model (see Figure 3d) and transform them into surface meshes of triangular elements; then, the surface meshes were converted into ten node tetrahedral elements (C3D10M), as shown in Figure 4a. Grid refinement was applied to the thinner cell walls (see the subfigure of Figure 4a) so that the wall thickness direction contains at least two layers of grids to ensure calculation accuracy. The entire 3D rRVE has a total of 6,661,478 elements, as shown in Figure 4b. Fix the six degrees of freedom of the bottom base and retain 1 degree of freedom of the upper plate in the compression direction. Finally, a sequential thermal–mechanical coupling analysis was adopted, i.e., applying temperature boundary conditions and then applying a compression displacement of 1.5 mm to the rRVE, as illustrated in Figure 4b.

When polymers are compressed, yielding and plastic deformation often occur [29]. The heat distortion temperature and glass transition temperature (T_g_) of PMI matrix material are usually higher than 150 °C, and this means that PMI foam may exhibit elastoplastic behavior at 20–120 °C and low strain rates (0.00067/s). Therefore, this work chooses the commonly used Ductile failure criterion to describe the failure process of PMI foam and uses yield stress softening and stiffness reduction to achieve the failure analysis. The damage variable $\dot{D}$ is defined as:

$$\dot{D}=\frac{\dot{{\bar{\varepsilon }}^{pl}}-{\bar{\varepsilon }}_{0}^{pl}}{{\bar{\varepsilon }}_{f}^{pl}-{\bar{\varepsilon }}_{0}^{pl}}\left(\dot{{\bar{\varepsilon }}^{pl}}\geq {\bar{\varepsilon }}_{0}^{pl}\right)$$

where, $\dot{{\bar{\varepsilon }}^{pl}}$, ${\bar{\varepsilon }}_{0}^{pl}$, and ${\bar{\varepsilon }}_{f}^{pl}$ represent the equivalent plastic strain, the fracture strain, and the final failure equivalent plastic strain, respectively. In addition, *D* = 0 indicates no damage; 0 < *D* < 1 denotes the occurrence of damage, and *D* = 1 represents complete damage. The stiffness of PMI matrix material is reduced in a proportion of 1 − *D*. During the simulation, the required parameters, such as elastic modulus, yield stress, and yield strain of the PMI matrix, are listed in Figure 4c (provided by the manufacturer).

## 3. Results and Discussion

### 3.1. DMA and TGA Results

**Figure 5:**
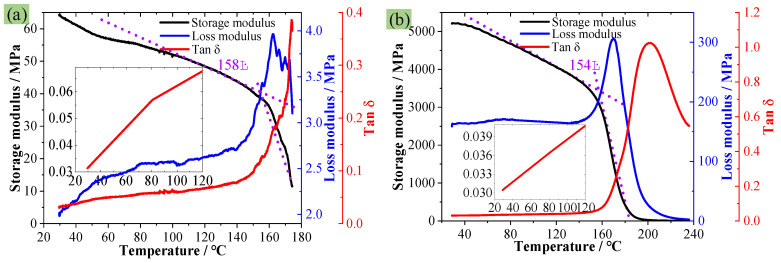
DMA experimental results, (**a**) PMI foam, (**b**) PMI matrix material.

Figure 5a and Figure 5b, respectively, present the DMA experimental results of PMI foam and PMI matrix (the curves are incomplete because of the softening of samples at around 180 °C). Figure 5 shows that the T_g_ of PMI foam and PMI matrix are measured as 158 °C and 154 °C, respectively. In addition, within the range of 20–120 °C, the loss factor (Tanδ) of PMI foam and PMI matrix increases by about 0.04 and 0.008, while the loss modulus changes by 65.5% and 4.7%, respectively. This indicates that the viscosity of PMI changes nonsignificantly within the 20–120 °C range. Especially for the PMI matrix, the slight changes in Tanδ and loss modulus suggest that the PMI matrix can be considered an elastoplastic material in the range of 20–120 °C and at low strain rates (0.00067/s). However, the compression deformation or failure of PMI foam is attributed to the yield or failure of the PMI matrix. Thus, in the subsequent investigations, the PMI matrix is considered an elastoplastic material within the range of 20–120 °C.

**Figure 6:**
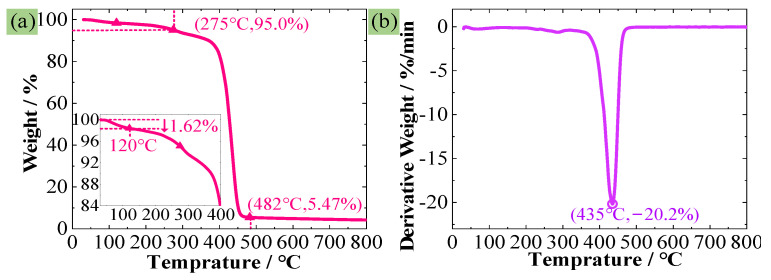
TGA experimental results, (**a**) TG curve, (**b**) DTG curve.

Figure 6a,b represent the TG curve and DTG curve of the PMI foam, respectively. As observed from Figure 6a and the subfigure, the mass loss at 120 °C is merely about 1.62%. If the temperature corresponding to a 5% mass loss is considered as the initial decomposition temperature, then the initial decomposition temperature determined according to Figure 6a is approximately 275 °C. Below 275 °C, the mass loss mainly results from the evaporation of water in PMI foam. When the temperature reaches about 400 °C, the PMI foam decomposes rapidly and basically completely decomposes at about 480 °C. As can be seen in Figure 6a, after 482 °C, the mass of coke residue is only 5.47% of the original mass, and it remains basically unchanged as the temperature further increases. Figure 6b reveals that the PMI foam exhibits a single thermal degradation peak, indicating a one-step thermal degradation process within the range of 360–480 °C, with a maximum degradation rate of −20.2%/min occurring at 435 °C. Therefore, Figure 6 further proves that PMI foam is thermally stable in the range of 20–120 °C.

### 3.2. Compressive Mechanical Behavior of PMI Foam at 20–120 °C

**Figure 7:**
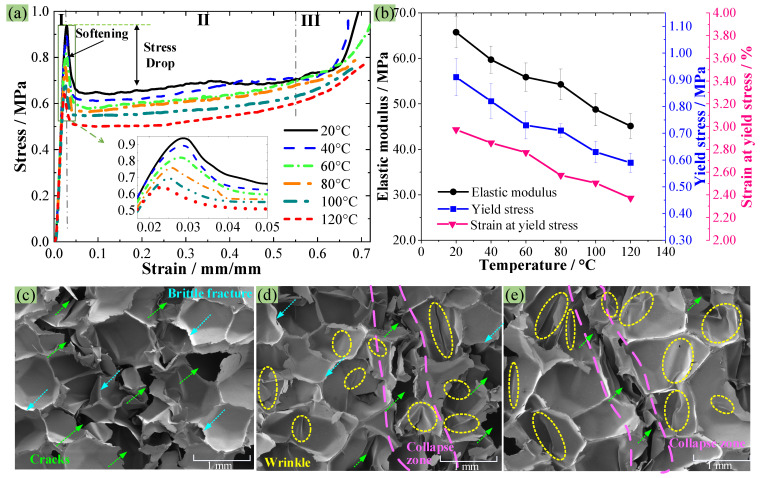
(**a**) Compressive stress–strain curves at different temperatures, (**b**) the obtained maximum yield stress, elastic modulus, and strain corresponding to the maximum yield stress, (**c**–**e**) SEM images of specimens failed at 20/80/120 °C.

Figure 7a gives the stress–strain curves of PMI foam at 20–120 °C, and all curves exhibit a “three-stage” pattern, i.e., an elastic stage (I), a plateau stage (II), and a densification stage (III). In addition, the subfigure in Figure 7a shows that the maximum yield stress (σ_s_) decreases as the temperature increases, and a sudden drop in stress occurs after reaching the σ_s_. The obtained compressive elastic modulus (*E*), σ_s_, and strain corresponding to σ_s_ (ε_s_) are given in Figure 7b. At 20 °C, the σ_s_ and *E* of PMI foam are 0.91 MPa and 65.8 MPa, respectively. While when the temperature increases to 40, 60, 80, 100, and 120 °C, the average σ_s_ and *E* decrease by 9.9% and 9.3%, 19.8% and 15.1%, 22.0% and 17.5%, 30.8% and 25.9%, and 35.2% and 31.4%, respectively. This is because the increasing temperature enhances the mobility and plastic deformation of PMI, thus leading to a reduction in σ_s_ and *E*. Moreover, the ε_s_ gradually decrease with increasing temperature, from 2.97% at 20 °C to 2.37% at 120 °C, indicating a 20.2% decrease at 120 °C. Therefore, during the molding process, the compression amount at each temperature should be controlled in a reasonable range to ensure the quality of the molded product. In addition, Figure 7b also shows that the σ_s_ and *E* within 60–80 °C decrease relatively slowly, which is similar to the results reported by Lv et al. [14], possibly due to the change in the failure mechanism of the PMI matrix caused by increasing temperature (A detailed discussion will be provided in Section 3.4).

Figure 7c–e presents the SEM images of the compressive collapse zone of PMI foam at 20, 80, and 120 °C, respectively. Figure 7c shows that at 20 °C, there are many continuous cracks in the failure area, and there are almost no obvious wrinkles on the surface of the crushed PMI cell walls. In addition, the fractured cross-sections of PMI cell walls are relatively smooth, indicating that the PMI tends to brittle failure at 20 °C; that is, the PMI foam belongs to elastic–brittle rigid foams at room temperature [30]. When the temperature rises to 80 °C (Figure 7d), many cell walls are pressed into wrinkles, which indicates that the failure mode of PMI foam changes to plastic deformation/yield of the PMI matrix. When the temperature reaches 120 °C, as shown in Figure 7e, a large number of wrinkles appear on the cell walls, suggesting that the failure mode of PMI foam at 120 °C is mainly dominated by the plastic yield of PMI matrix.

### 3.3. Failure Mechanism of PMI Foam under Thermal–Mechanical Coupling

**Figure 8:**
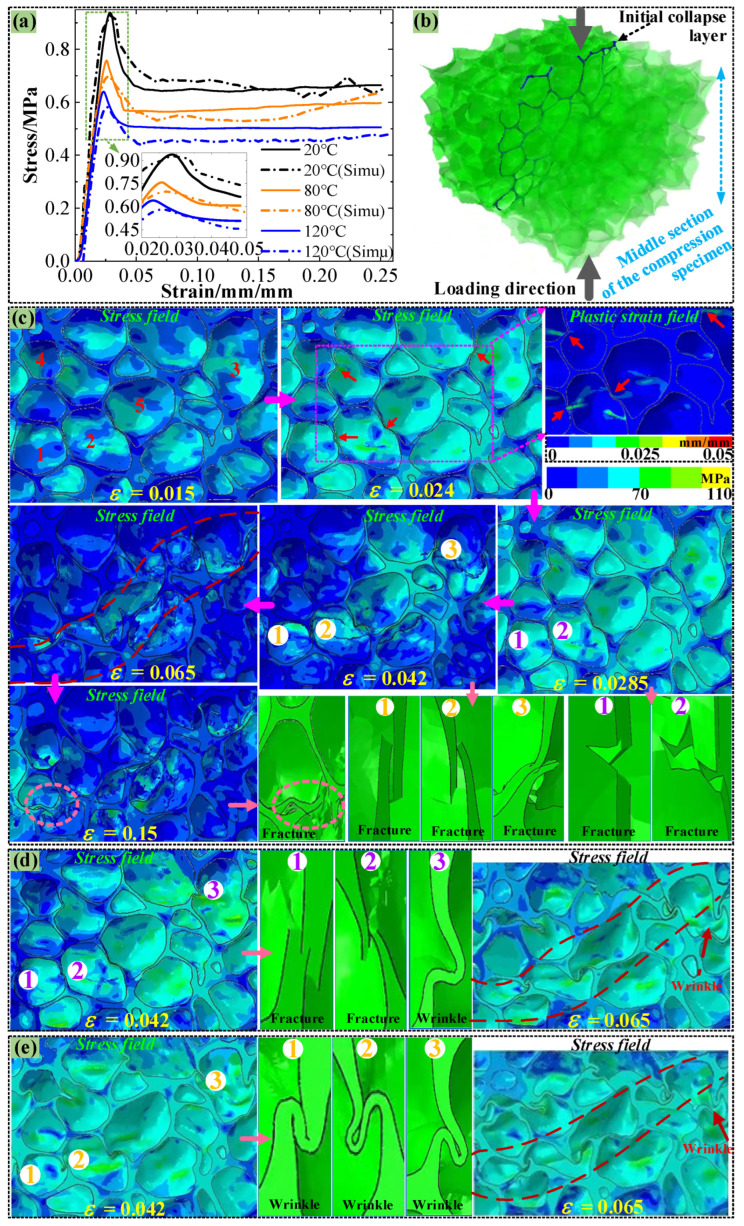
(**a**) Simulated stress–strain curves and experimental curves, (**b**) simulated initial collapse zone at 20 °C, Numbers 1–5: Cell numbering, Red dashed line: shape of collapsed area, (**c**) deformation process of rRVE at 20 °C, (**d**) deformation process during the stress drop stage at 80 °C, (**e**) deformation process during the stress drop stage at 120 °C.

Based on the numerical model established in Figure 4, the compressive stress–strain curves of PMI foam at 20, 80, and 120 °C are obtained and given in Figure 8a, which are compared with the experimental curves. It can be seen that at 20 °C, the simulated σ_s_ value is very close to the experimental value, but at 80 °C and 120 °C, the simulated σ_s_ values are slightly lower than the experimental results (see the subfigure in Figure 8a, and the detailed reason will be given in Section 3.4). Because some local cell walls of PMI foam are thin, the cells with thinner wall thickness in the compression direction would first lose stability and form an initial collapse zone, as illustrated in Figure 8b, which is the collapse zone formed when PMI foam is compressed at 20 °C.

Simultaneously, Figure 8c presents the stress field distribution of PMI foam under different strains during the compression at 20 °C. When the strain (ε) is 0.015, the cell walls undergo elastic compression, resulting in a relatively uniform stress field (see those labeled cells 1–5). As the ε increases to 0.024, the stress field distributes more uniformly, and a local equivalent plastic strain field shows that the walls of cells 1–5 undergo certain plastic deformation (ε^P^ < 0.02), which will reduce the load-bearing capacity of the foam cells and lead to a decrease in the slope of the stress–strain curve. When the ε rises to 0.0285, the compressive stress reaches σ_s_, and the cells 1–2 first rupture. As the ε increases to 0.042, the rapid collapse of some cell walls leads to the release of compressive stress in the surrounding area, thus resulting in a stress drop phenomenon (see the stress drop in Figure 8a). When the ε reaches 0.065, a macroscopic collapse zone is formed due to the complete collapse of some adjacent cells. Afterward, until the ε increases to 0.15, deformation or failure always concentrates in the collapse zone and extends to both sides, corresponding to the “plateau stage” of the stress–strain curve, which is consistent with the SEM result shown in Figure 7c.

Figure 8d,e present some stress fields and corresponding local deformation/failure of PMI foam during the compression at 80 and 120 °C. As shown in Figure 8d, during the stress drop stage at 80 °C (ε = 0.042), some cells (As indicated by the arrow) still fail brittlely, while others show a significant plastic deformation feature (wrinkles). Thus, due to the appearance of obvious plastic yielding, the collapse speed of foam cells is slowed down, which leads to a reduction in stress drop. When the ε reaches 0.065, the damaged cells are crushed, and some plastic-damaged cells have clear and obvious wrinkled boundaries, which is consistent with the SEM result shown in Figure 7d. In fact, at 120 °C, as shown in Figure 8e, during the stress drop stage (ε = 0.042), no brittle fractured cells are observed, while most foam cells exhibit obvious plastic failure (many wrinkles), and this will significantly reduce the degree of stress drop. When the cells are completely crushed (ε = 0.065), many wrinkles and a significant collapse zone can be clearly observed (as indicated by the arrows), which is consistent with the SEM result given in Figure 7e.

### 3.4. Influence of Air in Foam Cells on the Compressive Behavior

**Figure 9:**
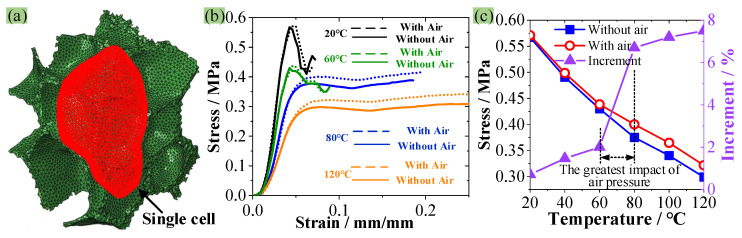
(**a**) Finite element model of a single cell under compression, (**b**) Simulation results, (**c**) Influence of gas on the single cell.

Figure 7b shows that the reduction rate of the σ_s_ and *E* of PMI foam within 60–80 °C is significantly lower than that in other temperature ranges, which may be due to the influence of air in foam cells. According to the foaming principle of polymer foams [31], PMI single closed cells are inevitably filled with air. Fahlbusch et al. [32] found that at room temperature and under quasi-static compression, the influence of air in foam cavities on foam mechanical properties is less than 1%, which can be neglected. Therefore, the foam cell labeled as 5 in Figure 8c is used to establish an RVE only containing a complete cell, as shown in Figure 9a, and the cell is treated as a surface-based fluid cavity to apply gas pressure [33]. In addition, only the degree of freedom of the top surface and four sides of the RVE in the compression direction is retained to prevent the lateral expansion of the foam [34]. The simulation is conducted at temperatures of 20, 60, 80, and 120 °C, and the calculations at each temperature are divided into two cases: with air and without air. In addition, since the air pressure in closed-cell foam is close to atmospheric pressure at 20 °C, the air pressure inside and outside the cell is set to 0.1 MPa. While at 60, 80, and 120 °C, the air pressure inside and outside the cell is calculated using the ideal gas state equation *pV* = *nRT*, where *p*, *V*, *n*, *R*, and *T* represent the pressure, volume, mass, gas constant and the absolute temperature of the air, respectively. The simulation calculation should be terminated when air leakage occurs with the destruction of foam cells.

Figure 9b illustrates the influence of air on the compressive mechanical behavior of PMI foam at different temperatures. The obtained σ_s_ is much lower than the experimental value, which is attributed to the stress reduction caused by the small size of the RVE model (Figure 9a). It can be seen that the presence or absence of air within the single cell has a small effect on the compressive stress–strain curves of PMI foam within the range of 20–60 °C. However, when the temperature rises to 80 °C, the influence of air pressure on the compressive stress of PMI foam increases, and the σ_s_ with air is about 6.7% higher than that without air. Similarly, at 120 °C, the σ_s_ with air improves by 7.5% than that without air. The influences of air pressure on the σ_s_ of PMI foam, as well as the increment of σ_s_ with air compared with σ_s_ without air at different temperatures, are presented in Figure 9c. It can be seen that after 60 °C, the influence of air pressure on the σ_s_ becomes more obvious. In addition, this influence is most significant in the 60–80 °C range. This is because, in the range of 60–80 °C, the failure mode of PMI foam begins to change to plastic yield. The delayed destruction of foam cells increases the pressure inside the cells, thereby increasing the compressive capacity of PMI foam. Therefore, within the range of 60–80 °C, the decrease rate in σ_s_ and *E* obtained from the experiment decreases obviously (see Figure 7b). However, when the temperature exceeds 80 °C, because the plastic deformation of PMI foam increases with the temperature, the pressure in the cells cannot always increase significantly, so the influence of air does not increase with the further increased temperature.

## 4. Conclusions

The compressive mechanical properties and failure mechanism of PMI foam in the range of 20–120 °C are investigated systematically. First, DMA experiment results show that PMI foam can be considered as an elastoplastic material due to lower mechanical loss within 20–120 °C and higher T_g_ of 158 °C, and TGA result proves the thermal stability of PMI foam within 20–120 °C. Second, the compression tests indicate that the σ_s_ of PMI foam at 80 °C and 120 °C, respectively, decrease by 22.0% and 35.2% compared with that at 20 °C, while the *E* decreases by 17.5% and 31.4%, respectively. In addition, when the temperature is lower than 80 °C, PMI foam mainly exhibits brittle fracture while changing to plastic yield failure after 80 °C. Third, the simulations based on rRVE reveal that the PMI cell walls mainly undergo brittle fracture at 20 °C, while at 80 °C, both brittle fracture and plastic yield occur. When the temperature reaches 120 °C, most cell walls undergo plastic yield failure. Thus, the temperature zone near 80 °C may be the transition zone of brittle–plastic failure of PMI foam. Finally, the simulation based on a single-cell RVE shows that the air pressure in the PMI foam cells has a significant effect on the compressive mechanical behavior of PMI foam when the temperature rises to 60 °C, and the effect can reach 6.7% at 80 °C, which cannot be ignored. Therefore, this study reveals that during the molding process of polymer foam sandwich structure, temperature, and pressure will significantly affect the compression performance and failure mechanism of PMI foam.

## Data Availability

Data are contained within the article.
